# (*Z*)-3-Chloro-3-phenyl-*N*-[(*S*)-1-phenyl­ethyl]prop-2-enamide

**DOI:** 10.1107/S160053680801876X

**Published:** 2008-07-05

**Authors:** Neudo A. Urdaneta, Teresa González, Alexander Briceño

**Affiliations:** a Laboratorio de Organica 210, Departamento de Química, Universidad Simon Bolivar, Apartado 47206, Caracas 1080-A, Venezuela; b Centro de Química, Instituto Venezolano de Investigaciones Científica (IVIC), Apartado 21827, Caracas 1020-A, Venezuela

## Abstract

The asymmetric unit of the title compound, C_17_H_16_ClNO, contains two crystallographically independent mol­ecules. These mol­ecules are connected in an alternating fashion through N—H⋯O and C—H⋯O hydrogen bonds, generating one-dimensional chains of graph sets *R*_2_^1^(6) and *C*(4) along the *a* axis.

## Related literature

For related literature, see: Kishikawa *et al.*, (1997 ▶); Cherry *et al.* (2003 ▶); Pontiki & Hadjipavlou (2007 ▶); Urdaneta *et al.* (2004 ▶). For graph-set notation, see: Bernstein *et al.* (1995 ▶).
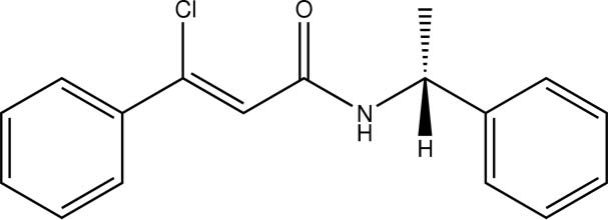


## Experimental

### 

#### Crystal data

C_17_H_16_ClNO
*M* *_r_* = 285.76
Orthorhombic, 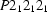

*a* = 9.803 (3) Å
*b* = 14.976 (5) Å
*c* = 20.823 (6) Å
*V* = 3057.2 (15) Å^3^
*Z* = 8
Mo *K*α radiation
μ = 0.24 mm^−1^
*T* = 293 (2) K
0.48 × 0.38 × 0.28 mm

#### Data collection

Rigaku AFC-7S Mercury diffractometer
Absorption correction: multi-scan (Jacobson, 1998 ▶) *T*_min_ = 0.897, *T*_max_ = 0.985 (expected range = 0.850–0.934)
32660 measured reflections
5802 independent reflections
3687 reflections with *I* > 2σ(*I*)
*R*_int_ = 0.063

#### Refinement

*R*[*F* ^2^ > 2σ(*F* ^2^)] = 0.070
*wR*(*F* ^2^) = 0.161
*S* = 1.07
5802 reflections
362 parameters
H-atom parameters constrained
Δρ_max_ = 0.18 e Å^−3^
Δρ_min_ = −0.28 e Å^−3^
Absolute structure: Flack (1983 ▶), 1693 Friedel pairs
Flack parameter: −0.03 (9)

### 

Data collection: *CrystalClear* (Rigaku, 2002 ▶); cell refinement: *CrystalClear*; data reduction: *CrystalStructure* (Rigaku/MSC, 2004 ▶); program(s) used to solve structure: *SHELXTL-NT* (Sheldrick, 2008 ▶); program(s) used to refine structure: *SHELXTL-NT*; molecular graphics: *SHELXTL-NT* and *DIAMOND* (Brandenburg, 1999 ▶); software used to prepare material for publication: *SHELXTL-NT* and *PLATON* (Spek, 2003 ▶).

## Supplementary Material

Crystal structure: contains datablocks global, I. DOI: 10.1107/S160053680801876X/hb2746sup1.cif

Structure factors: contains datablocks I. DOI: 10.1107/S160053680801876X/hb2746Isup2.hkl

Additional supplementary materials:  crystallographic information; 3D view; checkCIF report

## Figures and Tables

**Table 1:** Hydrogen-bond geometry (Å, °)

*D*—H⋯*A*	*D*—H	H⋯*A*	*D*⋯*A*	*D*—H⋯*A*
N1—H1*N*⋯O2^i^	0.97	1.89	2.852 (4)	174
N2—H2*N*⋯O1	0.95	2.04	2.933 (5)	157
C10—H10⋯Cl1	0.93	2.64	3.021 (6)	105
C13—H13⋯N1	0.93	2.55	2.874 (5)	101
C19—H19⋯O1	0.93	2.50	3.315 (5)	146
C27—H27⋯Cl2	0.93	2.65	3.028 (6)	105
C30—H30⋯N2	0.93	2.65	2.951 (5)	99

Symmetry code: (i) 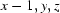.

**Fig. 1.**
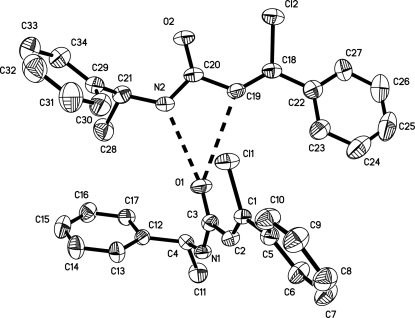
The molecular structure of (I) with displacement ellipsoids drawn at the 30% probability level. H atoms have been omited for clarity and dashed lines indicate the donor···acceptor interactions for the hydrogen bonds.

**Fig. 2.**
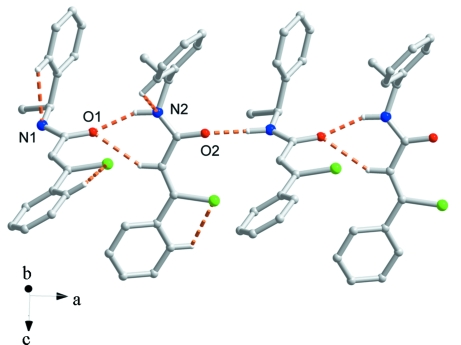
View of the one-dimensional ribbons along the a axis, generated by intermolecular hydrogen bonds. Intramolecular hydrogen bonds are also shown (dashed lines). Most H atoms have been omited for clarity

## supplementary crystallographic information

### Comment

The title compound, (I), represents a valuable intermediate for the synthesis of biologically active disubstituted pyrimidones (Cherry *et al.*, 2003), phenyl-substituted amides with antioxidant and anti-inflamatory activity as novel lipoxygenase inhibitor (Pontiki & Hadjipavlou, 2007), and also as precursor for photochemical studies (Kishikawa *et al.*, 1997).

The asymmetric unit of (I) contains two crystallographically indepedent molecules of the same stereochemical configuration (Fig. 1): C4 and C21 have S configuration.  Each molecule displays two kinds of intramolecular C—H···Cl and C—H···N hydrogen bonds (Table 1). These interactions lead to the formation of five-membered rings described by graph-set symbol S(5) (Bernstein *et al.*, 1995). In each molecule the phenyl groups are twisted with respect to the aliphatic chain defined by C4/N1/C3/O1/C2/C1 (CH1) and C21/N2/C18/C19/C20/O2 atoms (CH2), respectively. The dihedral angles between the C5—C10 and C12—C17 rings and the mean plane of the CH1 are 31.8 (2)° and 88.6 (2)°, for the molecule 1; C29—C34: 81.8 (2)° and C22—C2: 33.8 (2)° for the rings of the molecule 2. These molecules form a dimer linked through a N—H···O and C—H···O intermolecular hydrogen bonds in which the O atom from carbonyl group acts as a double aceptor of hydrogen bonds (Fig 1). This interaction produces a supramolecular motif described by the symbol *R*_2_^1^(6). These dimers are connected in an alternate fashion *via* remaining N—H···O intermolecular hydrogen bonds, generating one-dimensional chains along the *a* axis (Fig. 2), this interaction is described by the symbol C(4). Adjacent chains are assembled through C—H···π interactions to afford a three-dimensional array.

### Experimental

The title compound was prepared according to a reported procedure (Urdaneta *et al.*, 2004), and colourless blocks of (I) were grown from a saturated AcOEt/Et_2_O (1:9) solution kept at 277 K.

### Refinement

The N-bound H atoms were located in difference maps and refined as riding in their as-found relative positions with U_iso_(H) = 1.2U_eq_(N). The C-bound H atoms were placed in idealised positions (C—H = 0.93-0.98Å) and refined as riding with *U*_iso_(H) = 1.2U_eq_(C) or 1.5U_eq_(methyl C).

### Crystal data

**Table:**

C_17_H_16_ClNO	*F*_000_ = 1200
*M**_r_* = 285.76	*D*_x_ = 1.242 Mg m^−^^3^
Orthorhombic, *P*2_1_2_1_2_1_	Mo *K*α radiation λ = 0.71070 Å
Hall symbol: P 2ac 2ab	Cell parameters from 16200 reflections
*a* = 9.803 (3) Å	θ = 1.7–27.5º
*b* = 14.976 (5) Å	µ = 0.25 mm^−^^1^
*c* = 20.823 (6) Å	*T* = 293 (2) K
*V* = 3057.2 (15) Å^3^	Block, colourless
*Z* = 8	0.48 × 0.38 × 0.28 mm

### Data collection

**Table:**

Rigaku AFC-7S Mercury diffractometer	5802 independent reflections
Radiation source: Normal-focus sealed tube	3687 reflections with *I* > 2σ(*I*)
Monochromator: graphite	*R*_int_ = 0.063
*T* = 293(2) K	θ_max_ = 28.0º
ω scans	θ_min_ = 1.7º
Absorption correction: multi-scan(Jacobson, 1998)	*h* = −8→11
*T*_min_ = 0.897, *T*_max_ = 0.985	*k* = −17→17
32660 measured reflections	*l* = −24→24

### Refinement

**Table:**

Refinement on *F*^2^	H-atom parameters constrained
Least-squares matrix: full	*w* = 1/[σ^2^(*F*_o_^2^) + (0.0533*P*)^2^ + 1.3975*P*] where *P* = (*F*_o_^2^ + 2*F*_c_^2^)/3
*R*[*F*^2^ > 2σ(*F*^2^)] = 0.070	(Δ/σ)_max_ < 0.001
*wR*(*F*^2^) = 0.161	Δρ_max_ = 0.18 e Å^−^^3^
*S* = 1.07	Δρ_min_ = −0.28 e Å^−^^3^
5802 reflections	Extinction correction: SHELXLTL-NT (Sheldrick, 2008), Fc^*^=kFc[1+0.001xFc^2^λ^3^/sin(2θ)]^-1/4^
362 parameters	Extinction coefficient: 0.0040 (7)
Primary atom site location: structure-invariant direct methods	Absolute structure: Flack (1983), 1693 Friedel pairs
Secondary atom site location: difference Fourier map	Flack parameter: −0.03 (9)
Hydrogen site location: difmap and geom	

### Special details

**Table:**

Geometry. All e.s.d.'s (except the e.s.d. in the dihedral angle between two l.s. planes) are estimated using the full covariance matrix. The cell e.s.d.'s are taken into account individually in the estimation of e.s.d.'s in distances, angles and torsion angles; correlations between e.s.d.'s in cell parameters are only used when they are defined by crystal symmetry. An approximate (isotropic) treatment of cell e.s.d.'s is used for estimating e.s.d.'s involving l.s. planes.
Refinement. Refinement of *F*^2^ against ALL reflections. The weighted *R*-factor *wR* and goodness of fit *S* are based on *F*^2^, conventional *R*-factors *R* are based on *F*, with *F* set to zero for negative *F*^2^. The threshold expression of *F*^2^ > σ(*F*^2^) is used only for calculating *R*-factors(gt) *etc*. and is not relevant to the choice of reflections for refinement. *R*-factors based on *F*^2^ are statistically about twice as large as those based on *F*, and *R*- factors based on ALL data will be even larger.

### Fractional atomic coordinates and isotropic or equivalent isotropic displacement parameters (Å^2^)

**Table:**

	*x*	*y*	*z*	*U*_iso_*/*U*_eq_	
Cl1	0.22516 (10)	0.07828 (9)	0.67679 (6)	0.0689 (4)	
Cl2	0.67708 (13)	−0.13296 (11)	0.71017 (7)	0.0894 (5)	
O1	0.1409 (3)	−0.0600 (2)	0.58351 (14)	0.0657 (9)	
O2	0.6351 (3)	−0.0446 (3)	0.58420 (16)	0.0829 (11)	
N1	−0.0829 (3)	−0.0843 (2)	0.56625 (16)	0.0500 (9)	
H1N	−0.1799	−0.0749	0.5718	0.075*	
N2	0.4251 (4)	−0.0362 (3)	0.54294 (19)	0.0606 (10)	
H2N	0.3285	−0.0424	0.5436	0.091*	
C1	0.0512 (4)	0.0668 (3)	0.68930 (19)	0.0472 (10)	
C2	−0.0235 (4)	0.0157 (3)	0.6501 (2)	0.0545 (12)	
H2	−0.1172	0.0186	0.6567	0.065*	
C3	0.0199 (4)	−0.0450 (3)	0.5976 (2)	0.0530 (11)	
C4	−0.0595 (4)	−0.1488 (3)	0.5152 (2)	0.0505 (11)	
H4	0.0189	−0.1856	0.5275	0.061*	
C5	−0.0041 (4)	0.1173 (3)	0.7437 (2)	0.0559 (12)	
C6	−0.1234 (5)	0.0899 (3)	0.7744 (2)	0.0677 (13)	
H6	−0.1676	0.0382	0.7610	0.081*	
C7	−0.1763 (5)	0.1392 (4)	0.8244 (2)	0.0772 (15)	
H7	−0.2560	0.1202	0.8445	0.093*	
C8	−0.1139 (5)	0.2153 (4)	0.8451 (2)	0.0749 (15)	
H8	−0.1520	0.2489	0.8781	0.090*	
C9	0.0041 (5)	0.2415 (4)	0.8171 (2)	0.0771 (15)	
H9	0.0488	0.2921	0.8322	0.092*	
C10	0.0592 (5)	0.1940 (4)	0.7663 (3)	0.0736 (15)	
H10	0.1394	0.2137	0.7471	0.088*	
C11	−0.1834 (5)	−0.2102 (3)	0.5095 (3)	0.0742 (15)	
H11A	−0.2015	−0.2374	0.5503	0.111*	
H11B	−0.1652	−0.2558	0.4782	0.111*	
H11C	−0.2613	−0.1760	0.4963	0.111*	
C12	−0.0270 (4)	−0.1055 (3)	0.4513 (2)	0.0498 (11)	
C13	−0.0587 (5)	−0.0188 (3)	0.4370 (2)	0.0660 (13)	
H13	−0.1011	0.0165	0.4679	0.079*	
C14	−0.0291 (6)	0.0174 (4)	0.3779 (3)	0.0816 (16)	
H14	−0.0522	0.0762	0.3688	0.098*	
C15	0.0353 (6)	−0.0344 (4)	0.3321 (3)	0.0823 (17)	
H15	0.0554	−0.0101	0.2921	0.099*	
C16	0.0697 (5)	−0.1213 (4)	0.3450 (2)	0.0748 (15)	
H16	0.1129	−0.1561	0.3141	0.090*	
C17	0.0391 (4)	−0.1558 (3)	0.4044 (2)	0.0598 (13)	
H17	0.0633	−0.2145	0.4135	0.072*	
C18	0.5007 (4)	−0.1343 (3)	0.6992 (2)	0.0566 (12)	
C19	0.4457 (4)	−0.0998 (3)	0.6462 (2)	0.0562 (12)	
H19	0.3510	−0.1024	0.6444	0.067*	
C20	0.5118 (4)	−0.0577 (3)	0.5898 (2)	0.0564 (12)	
C21	0.4705 (5)	−0.0075 (4)	0.4790 (2)	0.0692 (14)	
H21	0.5526	−0.0416	0.4682	0.083*	
C22	0.4252 (5)	−0.1776 (3)	0.7526 (2)	0.0643 (13)	
C23	0.2911 (5)	−0.1517 (4)	0.7646 (2)	0.0753 (15)	
H23	0.2495	−0.1081	0.7395	0.090*	
C24	0.2199 (7)	−0.1923 (5)	0.8150 (3)	0.104 (2)	
H24	0.1321	−0.1733	0.8250	0.125*	
C25	0.2783 (10)	−0.2597 (6)	0.8497 (3)	0.118 (3)	
H25	0.2282	−0.2879	0.8817	0.141*	
C26	0.4087 (9)	−0.2858 (5)	0.8380 (3)	0.110 (2)	
H26	0.4480	−0.3314	0.8620	0.132*	
C27	0.4825 (6)	−0.2440 (4)	0.7900 (3)	0.0804 (16)	
H27	0.5725	−0.2608	0.7827	0.096*	
C28	0.3596 (6)	−0.0315 (4)	0.4295 (2)	0.0913 (18)	
H28A	0.3381	−0.0938	0.4328	0.137*	
H28B	0.3924	−0.0188	0.3870	0.137*	
H28C	0.2792	0.0032	0.4377	0.137*	
C29	0.5052 (5)	0.0901 (4)	0.4736 (2)	0.0669 (14)	
C30	0.4374 (6)	0.1545 (4)	0.5075 (3)	0.0836 (16)	
H30	0.3710	0.1374	0.5370	0.100*	
C31	0.4653 (8)	0.2447 (5)	0.4992 (4)	0.107 (2)	
H31	0.4184	0.2873	0.5231	0.128*	
C32	0.5621 (9)	0.2707 (5)	0.4559 (4)	0.113 (2)	
H32	0.5811	0.3310	0.4502	0.136*	
C33	0.6305 (7)	0.2085 (6)	0.4209 (4)	0.118 (3)	
H33	0.6956	0.2267	0.3912	0.142*	
C34	0.6043 (6)	0.1177 (5)	0.4292 (3)	0.0914 (19)	
H34	0.6523	0.0757	0.4053	0.110*	

### Atomic displacement parameters (Å^2^)

**Table:**

	*U*^11^	*U*^22^	*U*^33^	*U*^12^	*U*^13^	*U*^23^
Cl1	0.0419 (6)	0.0814 (9)	0.0833 (9)	−0.0033 (6)	0.0006 (5)	−0.0198 (7)
Cl2	0.0494 (7)	0.1156 (12)	0.1032 (11)	0.0039 (7)	−0.0093 (7)	0.0216 (9)
O1	0.0387 (17)	0.089 (3)	0.069 (2)	0.0026 (16)	0.0028 (14)	−0.0176 (18)
O2	0.0356 (18)	0.127 (3)	0.086 (2)	0.0029 (17)	0.0049 (16)	0.012 (2)
N1	0.0369 (18)	0.063 (2)	0.050 (2)	−0.0017 (17)	−0.0002 (15)	−0.0064 (19)
N2	0.0381 (19)	0.074 (3)	0.070 (3)	−0.0029 (18)	−0.0024 (18)	0.005 (2)
C1	0.035 (2)	0.058 (3)	0.049 (3)	−0.003 (2)	−0.0022 (18)	0.002 (2)
C2	0.042 (2)	0.063 (3)	0.059 (3)	0.002 (2)	0.006 (2)	0.005 (2)
C3	0.051 (3)	0.059 (3)	0.049 (3)	0.002 (2)	0.003 (2)	−0.002 (2)
C4	0.050 (2)	0.053 (3)	0.049 (3)	0.003 (2)	−0.007 (2)	0.003 (2)
C5	0.050 (2)	0.072 (3)	0.046 (3)	0.002 (2)	0.001 (2)	−0.004 (2)
C6	0.067 (3)	0.074 (4)	0.062 (3)	−0.011 (3)	0.017 (2)	−0.013 (3)
C7	0.068 (3)	0.099 (4)	0.065 (3)	−0.007 (3)	0.022 (3)	−0.015 (3)
C8	0.071 (3)	0.092 (4)	0.061 (3)	0.006 (3)	0.010 (3)	−0.023 (3)
C9	0.077 (3)	0.081 (4)	0.073 (4)	−0.010 (3)	0.013 (3)	−0.025 (3)
C10	0.059 (3)	0.085 (4)	0.077 (4)	−0.010 (3)	0.002 (3)	−0.019 (3)
C11	0.080 (3)	0.068 (4)	0.075 (3)	−0.023 (3)	−0.010 (3)	−0.003 (3)
C12	0.048 (2)	0.060 (3)	0.042 (3)	−0.004 (2)	−0.0019 (18)	−0.002 (2)
C13	0.086 (4)	0.056 (3)	0.056 (3)	0.004 (3)	0.003 (3)	0.006 (3)
C14	0.110 (5)	0.059 (4)	0.076 (4)	0.000 (3)	0.005 (3)	0.018 (3)
C15	0.103 (4)	0.086 (5)	0.058 (3)	−0.022 (3)	0.011 (3)	0.007 (3)
C16	0.085 (4)	0.087 (4)	0.053 (3)	−0.004 (3)	0.010 (3)	−0.009 (3)
C17	0.057 (3)	0.067 (3)	0.055 (3)	0.001 (2)	−0.001 (2)	−0.009 (3)
C18	0.042 (2)	0.062 (3)	0.066 (3)	0.004 (2)	0.000 (2)	−0.011 (3)
C19	0.039 (2)	0.068 (3)	0.061 (3)	−0.001 (2)	0.004 (2)	−0.002 (3)
C20	0.037 (2)	0.063 (3)	0.069 (3)	0.004 (2)	0.003 (2)	−0.005 (2)
C21	0.057 (3)	0.083 (4)	0.067 (3)	0.006 (3)	0.007 (2)	0.004 (3)
C22	0.065 (3)	0.067 (3)	0.060 (3)	−0.007 (3)	−0.001 (3)	−0.017 (3)
C23	0.069 (3)	0.083 (4)	0.073 (3)	−0.011 (3)	0.010 (3)	−0.008 (3)
C24	0.089 (4)	0.123 (6)	0.100 (5)	−0.024 (4)	0.035 (4)	−0.025 (5)
C25	0.155 (8)	0.132 (7)	0.066 (4)	−0.041 (6)	0.025 (5)	−0.001 (4)
C26	0.150 (7)	0.110 (6)	0.070 (4)	−0.006 (5)	0.011 (5)	0.012 (4)
C27	0.093 (4)	0.091 (4)	0.058 (3)	0.001 (3)	−0.006 (3)	0.005 (3)
C28	0.101 (4)	0.100 (5)	0.073 (4)	−0.022 (3)	−0.018 (3)	0.002 (3)
C29	0.050 (3)	0.088 (4)	0.062 (3)	−0.002 (3)	−0.005 (2)	0.009 (3)
C30	0.083 (4)	0.084 (5)	0.084 (4)	−0.003 (3)	0.006 (3)	0.007 (3)
C31	0.137 (6)	0.075 (5)	0.108 (5)	−0.003 (4)	0.003 (5)	0.011 (4)
C32	0.127 (6)	0.088 (6)	0.124 (7)	−0.021 (5)	−0.027 (5)	0.024 (5)
C33	0.098 (5)	0.124 (7)	0.133 (7)	−0.027 (5)	−0.004 (5)	0.062 (6)
C34	0.071 (4)	0.108 (5)	0.096 (5)	−0.003 (3)	0.012 (3)	0.019 (4)

### Geometric parameters (Å, °)

**Table:**

Cl1—C1	1.734 (4)	C15—C16	1.370 (7)
Cl2—C18	1.745 (4)	C15—H15	0.9300
O1—C3	1.242 (5)	C16—C17	1.374 (7)
O2—C20	1.231 (5)	C16—H16	0.9300
N1—C3	1.338 (5)	C17—H17	0.9300
N1—C4	1.455 (5)	C18—C19	1.332 (6)
N1—H1N	0.9677	C18—C22	1.484 (7)
N2—C20	1.333 (6)	C19—C20	1.483 (6)
N2—C21	1.469 (6)	C19—H19	0.9300
N2—H2N	0.9518	C21—C29	1.504 (8)
C1—C2	1.338 (6)	C21—C28	1.541 (7)
C1—C5	1.467 (6)	C21—H21	0.9800
C2—C3	1.483 (6)	C22—C27	1.382 (7)
C2—H2	0.9300	C22—C23	1.394 (7)
C4—C12	1.513 (6)	C23—C24	1.399 (8)
C4—C11	1.528 (6)	C23—H23	0.9300
C4—H4	0.9800	C24—C25	1.367 (10)
C5—C10	1.387 (7)	C24—H24	0.9300
C5—C6	1.394 (6)	C25—C26	1.359 (9)
C6—C7	1.378 (6)	C25—H25	0.9300
C6—H6	0.9300	C26—C27	1.385 (8)
C7—C8	1.363 (7)	C26—H26	0.9300
C7—H7	0.9300	C27—H27	0.9300
C8—C9	1.353 (7)	C28—H28A	0.9600
C8—H8	0.9300	C28—H28B	0.9600
C9—C10	1.385 (7)	C28—H28C	0.9600
C9—H9	0.9300	C29—C30	1.368 (7)
C10—H10	0.9300	C29—C34	1.404 (7)
C11—H11A	0.9600	C30—C31	1.390 (8)
C11—H11B	0.9600	C30—H30	0.9300
C11—H11C	0.9600	C31—C32	1.366 (10)
C12—C13	1.369 (6)	C31—H31	0.9300
C12—C17	1.394 (6)	C32—C33	1.359 (10)
C13—C14	1.376 (7)	C32—H32	0.9300
C13—H13	0.9300	C33—C34	1.394 (9)
C14—C15	1.382 (7)	C33—H33	0.9300
C14—H14	0.9300	C34—H34	0.9300
			
C3—N1—C4	122.0 (3)	C16—C17—C12	121.9 (5)
C3—N1—H1N	128.2	C16—C17—H17	119.0
C4—N1—H1N	109.8	C12—C17—H17	119.0
C20—N2—C21	122.8 (4)	C19—C18—C22	126.0 (4)
C20—N2—H2N	126.9	C19—C18—Cl2	120.3 (4)
C21—N2—H2N	110.1	C22—C18—Cl2	113.7 (4)
C2—C1—C5	124.4 (4)	C18—C19—C20	130.1 (4)
C2—C1—Cl1	120.2 (3)	C18—C19—H19	114.9
C5—C1—Cl1	115.4 (3)	C20—C19—H19	114.9
C1—C2—C3	130.1 (4)	O2—C20—N2	121.2 (4)
C1—C2—H2	115.0	O2—C20—C19	124.9 (4)
C3—C2—H2	115.0	N2—C20—C19	113.9 (4)
O1—C3—N1	121.6 (4)	N2—C21—C29	114.9 (4)
O1—C3—C2	124.0 (4)	N2—C21—C28	108.9 (4)
N1—C3—C2	114.4 (4)	C29—C21—C28	109.6 (4)
N1—C4—C12	113.0 (3)	N2—C21—H21	107.7
N1—C4—C11	109.3 (4)	C29—C21—H21	107.7
C12—C4—C11	110.9 (3)	C28—C21—H21	107.7
N1—C4—H4	107.8	C27—C22—C23	118.9 (5)
C12—C4—H4	107.8	C27—C22—C18	122.2 (5)
C11—C4—H4	107.8	C23—C22—C18	118.9 (5)
C10—C5—C6	117.7 (4)	C22—C23—C24	118.9 (6)
C10—C5—C1	121.5 (4)	C22—C23—H23	120.5
C6—C5—C1	120.8 (4)	C24—C23—H23	120.5
C7—C6—C5	120.3 (5)	C25—C24—C23	120.6 (6)
C7—C6—H6	119.9	C25—C24—H24	119.7
C5—C6—H6	119.9	C23—C24—H24	119.7
C8—C7—C6	121.2 (5)	C26—C25—C24	120.8 (7)
C8—C7—H7	119.4	C26—C25—H25	119.6
C6—C7—H7	119.4	C24—C25—H25	119.6
C9—C8—C7	119.4 (5)	C25—C26—C27	119.4 (7)
C9—C8—H8	120.3	C25—C26—H26	120.3
C7—C8—H8	120.3	C27—C26—H26	120.3
C8—C9—C10	120.9 (5)	C22—C27—C26	121.3 (6)
C8—C9—H9	119.6	C22—C27—H27	119.3
C10—C9—H9	119.6	C26—C27—H27	119.3
C9—C10—C5	120.6 (5)	C21—C28—H28A	109.5
C9—C10—H10	119.7	C21—C28—H28B	109.5
C5—C10—H10	119.7	H28A—C28—H28B	109.5
C4—C11—H11A	109.5	C21—C28—H28C	109.5
C4—C11—H11B	109.5	H28A—C28—H28C	109.5
H11A—C11—H11B	109.5	H28B—C28—H28C	109.5
C4—C11—H11C	109.5	C30—C29—C34	117.9 (6)
H11A—C11—H11C	109.5	C30—C29—C21	122.4 (5)
H11B—C11—H11C	109.5	C34—C29—C21	119.5 (5)
C13—C12—C17	117.8 (4)	C29—C30—C31	121.7 (6)
C13—C12—C4	123.4 (4)	C29—C30—H30	119.1
C17—C12—C4	118.8 (4)	C31—C30—H30	119.1
C12—C13—C14	121.3 (5)	C32—C31—C30	119.7 (7)
C12—C13—H13	119.3	C32—C31—H31	120.2
C14—C13—H13	119.3	C30—C31—H31	120.2
C13—C14—C15	119.6 (5)	C33—C32—C31	120.1 (7)
C13—C14—H14	120.2	C33—C32—H32	119.9
C15—C14—H14	120.2	C31—C32—H32	119.9
C16—C15—C14	120.6 (5)	C32—C33—C34	120.8 (7)
C16—C15—H15	119.7	C32—C33—H33	119.6
C14—C15—H15	119.7	C34—C33—H33	119.6
C15—C16—C17	118.7 (5)	C33—C34—C29	119.8 (7)
C15—C16—H16	120.6	C33—C34—H34	120.1
C17—C16—H16	120.6	C29—C34—H34	120.1

### Hydrogen-bond geometry (Å, °)

**Table:**

*D*—H···*A*	*D*—H	H···*A*	*D*···*A*	*D*—H···*A*
N1—H1N···O2^i^	0.97	1.89	2.852 (4)	174
N2—H2N···O1	0.95	2.04	2.933 (5)	157
C10—H10···Cl1	0.93	2.64	3.021 (6)	105
C13—H13···N1	0.93	2.55	2.874 (5)	101
C19—H19···O1	0.93	2.50	3.315 (5)	146
C27—H27···Cl2	0.93	2.65	3.028 (6)	105
C30—H30···N2	0.93	2.65	2.951 (5)	99

Symmetry codes: (i) *x*−1, *y*, *z*.
